# Editorial for the Special Issue on Advanced Interconnect and Packaging

**DOI:** 10.3390/mi14010171

**Published:** 2023-01-10

**Authors:** Wen-Sheng Zhao

**Affiliations:** Zhejiang Provincial Key Lab of Large-Scale Integrated Circuit Design, School of Electronics and Information, Hangzhou Dianzi University, Hangzhou 310018, China; wshzhao@hdu.edu.cn

Unlike transistors, the continuous downscaling of feature size in CMOS technology leads to a dramatic rise in interconnect resistivity and concomitant performance degradation. At nanoscale technology nodes, interconnect delay and reliability become the major bottlenecks faced by modern integrated circuits. To resolve these interconnect problems, various emerging technologies, including airgap, nanocarbon, optical, and through-silicon via (TSV), have been proposed and investigated. For example, by virtue of TSV technology, dies can be stacked to increase the integration density. More importantly, 3D integration and packaging also offer the most promising platform to implement “More-than-Moore” technologies, providing heterogenous materials and technologies on a single chip.

This Special Issue seeks to showcase research papers on new developments in advanced interconnect and packaging, i.e., on the design, modeling, fabrication, and reliability assessment of emerging interconnect and packaging technologies. Additionally, there are two interesting papers on carbon nanotube interconnects and interconnect reliability issues.

In particular, Liu et al. successfully realize the thermocompression bonding of Pt–Pt metal electrodes through process exploration and form a packaging interconnection that meets the requirements [1]. Sun et al. achieve low-temperature assembly by reflowing 13.5Sn–37.5Bi–45In–4Pb quaternary eutectic solder paste and a SAC 305 solder ball together at 140 °C for 5 min [2]. Liu et al. investigate the flow process of nano glass powder melted at a high temperature [3]. To reduce the wettability of the glass paste on the Au electrode, they grow a silicon dioxide isolation layer on the surface of golden lead via chemical vapor deposition. Wang et al. investigate the chip-level hermetic package for a high-temperature graphene pressure sensor and demonstrate that the combination of Cu–Sn and Au–Au is extremely suitable for hermetic packaging [4]. Wu et al. provide theoretical support for the application of thin coatings at high temperatures and in harsh environments [5].

Wang et al. study the electrical performance of graphene-based on-chip spiral inductors by virtue of a physics-based equivalent circuit model [6]. Kim et al. propose a novel interposer channel structure with vertical tabbed vias to achieve high-speed signaling in high-bandwidth memory and demonstrate that the proposed channel structure could reduce dynamic power consumption [7]. Kim designs noise suppression structures that generate an electromagnetic bandgap and studies the mechanism of the proposed structure based on dispersion analysis [8]. Zheng et al. study the average power handling capability of corrugated slow-wave transmission lines [9]. Du et al. develop a mathematical degradation model for evaluating the degradation of vacuum packaged MEMS sensors and perform a temperature-accelerated test of MEMS gyroscopes with different vacuums [10]. Zhao et al. introduce recent studies on the physics-based modeling of the electromigration aging of interconnects [11].

Pan et al. present a novel wideband bandpass filter based on the integration of a substrate-integrated waveguide and a spoof surface plasmon polariton [12]. Wei et al. present a new method for the analytical approximation of spatial current/field profiles of frequency-selective surfaces and demonstrate that the transmission line loss has little influence on the current distribution [13]. Bai et al. propose a broadband frequency-selective rasorber based on spoof surface plasmon polaritons [14]. Tian et al. investigate the deadbeat current controllers for an isolated bidirectional dual-active-bridge dc–dc converter, including in the peak current mode and middle current mode [15]. Kuo et al. develop a flexible blood oxygen sensing system with a 3 × 3 array and use a flip chip package to integrate the sensing chip [16]. Finally, Xu et al. review the advantages, recent developments, and dilemmas of carbon nanotube-based interconnects from the perspective of different interconnect lengths and through-silicon via applications [17].

To conclude, I would like to take this opportunity to thank all the authors for submitting their papers to this Special Issue, as well as the reviewers for the effort and time they expended to improve the quality of the published papers. I also want to recognize Mr. Dikies Zhang from the *Micromachines* publishing office for his endless assistance and help in disseminating this Special Issue.

In view of the success reached in terms of the number and quality of papers published, we plan to open a second Volume where we hope to continue the conversation regarding the latest advances in interconnect and packaging.

## References

[1] Liu J., Wang J., Li M., Zhang H. (2022). High quality Pt-Pt metal bonding for high temperature packaging. Micromachines.

[2] Sun L., Guo Z., Zhao X., Liu Y., Tu K., Liu Y. (2022). A new low-temperature solder assembly technique to replace eutectic Sn-Bi solder assembly. Micromachines.

[3] Liu Y., Chen J., Zheng G. (2022). Study on the wetting mechanism between hot-melt nano glass powder and different substrates. Micromachines.

[4] Wang J., Zhang H., Chen X., Li M. (2022). Hermetic packaging based on Cu-Sn and Au-Au dual bonding for high-temperature graphene pressure sensor. Micromachines.

[5] Wu C., Wang J., Liu X., Li M., Zhu Z., Qi Y. (2022). Au wire ball welding and its reliability test for high-temperature environment. Micromachines.

[6] Wang D.W., Yuan M.J., Dai J.Y., Zhao W.S. (2022). Electrical modeling and characterization of graphene-based on-chip spiral inductors. Micromachines.

[7] Kim H., Lee S., Song K., Shin Y., Park D., Park J., Cho J., Ahn S. (2022). A novel interposer channel structure with vertical tabbed vias to reduce far-end crosstalk for next-generation high-bandwidth memory. Micromachines.

[8] Kim Y. (2022). Design of power/ground noise suppression structures based on a dispersion analysis for packages and interposers with low-loss substrates. Micromachines.

[9] Zheng Z., Tang M., Zhang H., Mao J. (2022). Average power handling capability of corrugated slow-wave transmission lines. Micromachines.

[10] Du G., Dong X., Huang X., Su W., Zhang P. (2022). Reliability evaluation based on mathematical degradation model for vacuum packaged MEMS sensor. Micromachines.

[11] Zhao W.S., Zhang R., Wang D.W. (2022). Recent progress in physics-based modeling of electromigration in integrated circuit interconnects. Micromachines.

[12] Pan D., You B., Wen X., Li X. (2022). Wideband substrate integrated waveguide chip filter using spoof surface plasmon polariton. Micromachines.

[13] Wei S., Wen W. (2022). Antenna current calculation based on equivalent transmission line model. Micromachines.

[14] Bai J., Yang Q., Liang Y., Gao X. (2022). Broadband frequency selective rasorber based on spoof surface plamson polaritons. Micromachines.

[15] Tian C., Wei S., Xie J., Bai T. (2022). Dynamic enhancement for dual active bridge converter with a deadbeat current controller. Micromachines.

[16] Kuo W.C., Wu T.C., Wang J.S. (2022). Design and application of a flexible blood oxygen sensing array for wearable devices. Micromachines.

[17] Xu B., Chen R., Zhou J., Liang J. (2022). Recent progress and challenges regarding carbon nanotube on-chip interconnects. Micromachines.

